# Race and Street-Level Firework Legalization as Primary Determinants of July 4th Air Pollution across Southern California

**DOI:** 10.3390/atmos14020401

**Published:** 2023-02-19

**Authors:** Shahir Masri, Leonel Flores, Jose Rea, Jun Wu

**Affiliations:** 1 Department of Environmental and Occupational Health, Program in Public Health, University of California, Irvine, CA 92697, USA; 2 Madison Park Neighborhood Association, GREEN-MPNA Programs, Santa Ana, CA 92707, USA

**Keywords:** firework, PM_2.5_, air pollution, citizen science, environmental justice

## Abstract

Air pollution is a major public health threat that is associated with asthma, cardiovascular disease, respiratory disease and all-cause mortality. Among the most important acute air pollution events occurring each year are celebrations involving fireworks, such as the 4th of July holiday in the United States. In this community-engaged study, academic partners and residents collaborated to collect indoor and outdoor PM_2.5_ concentration measurements in the disadvantaged city of Santa Ana, California, using low-cost AtmoTube sensor devices before, during and after the July 4th firework celebration, while also examining July 4th data extracted from the PurpleAir sensor network across over a hundred other cities in southern California. Average outdoor PM_2.5_ concentrations on July 4th were found to be three-to-five times higher than baseline, with hourly concentrations exceeding 160 μg/m^3^. Outdoor averages were roughly 30% to 100% higher than indoor levels. The most polluted cities exhibited 15-times higher PM_2.5_ levels compared with the least contaminated cities and were often those where household-level fireworks were legal for sale and use. Race/ethnicity was found to be the leading predictor of July 4th-related air pollution across three counties in southern California, with greater PM_2.5_ being associated with higher proportions of Hispanic residents and lower proportions of White residents. The findings from this study underscore the importance of environmental justice as it relates to firework-related air pollution exposure, and the critical role city- and county-level firework policies play in determining exposure.

## 1. Introduction

Air pollution presents a major threat to public health and has been widely associated with adverse health effects, such as asthma, cardiovascular disease and respiratory disease, along with all-cause mortality and hospital admissions [1–5]. In a recent study analyzing “urban-associated diseases” including allergies, asthma and cancer, air pollution was demonstrated to be the urban factor most frequently associated with adverse health effects [6]. Particulate matter with an aerodynamic diameter of less than 2.5 μm (PM_2.5_) is especially harmful to health, having been associated with cardiovascular disease, lung cancer and preterm birth [7–9] and contributing to over eight million deaths per year [10]. In a 2019 study of the Global Burden of Disease report, Yang et al. (2021) showed a substantial increase in the number of deaths from 1990 to 2019 due to chronic obstructive pulmonary disease (COPD) attributable to PM_2.5_ exposure [11].

In the United States, extensive literature has similarly shown low-income communities and communities of color to incur a disproportionate amount of exposure to air pollutants and other environmental hazards both in California and nationally [12–20]. Specifically, Tessum et al. (2021) reported disproportionate PM_2.5_ exposure among people of color that was systematically evident across nearly all major emission categories and was consistent across states, urban and rural areas, income levels, and exposure levels [21]. Importantly, while air pollution trends have generally improved over time throughout the U.S., disparities in PM_2.5_ and NO_2_ exposure have increased in some regions, highlighting the ongoing challenges regarding air pollution and related equity [22].

Among the most important acute air pollution events occurring each year are holiday celebrations involving the use of fireworks [23–26], with the 4th of July and New Year’s Eve being the most salient such holidays in the United States [23,27]. During (and/or shortly before and after) festivals and national holidays, daily PM_2.5_ concentrations have been documented to be two-to-ten times higher than baseline levels [27,28]. Importantly, results from toxicological studies have found particles generated by fireworks to produce deleterious effects in mammalian cells and lungs, highlighting the need to further investigate the contribution of firework-related emissions in the context of public health [25,29]. Air pollution generated by fireworks and other sources can be either exacerbated or alleviated by the effects of photochemistry and local meteorology, including wind speed, wind direction, planetary boundary layer height, atmospheric stability, and precipitation [30].

To date, the majority of air pollution studies related to firework celebrations have focused on characterizing the magnitude and composition of firework pollution, and usually do so at a low spatial resolution and only in the outdoor environment. Seidel et al. (2015), for instance, examined hourly and daily PM_2.5_ measurements across the U.S. on July 4th using sparsely distributed outdoor air quality monitoring operated by the U.S. Environmental Protection Agency (EPA), while Dickerson et al. (2017) conducted a similar large-scale firework-related study using sparse outdoor EPA monitors, albeit focusing on chemical species. While previous studies have shown firework episodes to increase regional PM_2.5_ concentrations, their common use of sparely distributed monitoring stations means that measurements often fall outside of urban population centers that are most relevant to exposure [29,31–35]. Investigations of the indoor environment during firework episodes at the residential scale are therefore needed to better understand firework-related personal exposure for those who remain either indoors or outdoors during such periods.

Previously, we made use of low-cost air pollution sensors to quantify July 4th firework-related PM_2.5_ at the census tract level in California and the influence of the 2020 pandemic period [36]. While we found associations between socioeconomic factors and air pollution, this study examined only outdoor PM_2.5_ concentrations and considered neither the variability of such patterns within counties nor the influence of firework legalization at the city level. Currently, a research gap exists as it relates to better understanding firework-related air pollution in the context of socioeconomic factors and related differences in the legalization of street-level fireworks.

Low-cost air pollution sensors have increasingly been used by experts to map air contamination at a high spatial and temporal resolution [37–40]. This has improved upon traditional government-operated monitoring stations which often lack the spatial resolution needed to characterize community-level air pollution hotspots such as those created by wildfires, firework celebrations, etc. [36,40–42]. Moreover, their low cost, mobility and ease of maintenance has enabled community-engaged participatory research through which trained residents, including racially marginalized groups, are able to participate in the development of research aims, data collection and the dissemination of results [43–48].

In this study, the Madison Park Neighborhood Association (MPNA) non-profit known as GREEN-MPNA engaged community and academic partners in order to characterize July 4th firework-related air pollution both indoors and outdoors at the neighborhood level in Santa Ana, California, and at the city level throughout southern California, using low-cost air pollution sensors. Our specific aims included: (1) characterizing the temporal pattern of outdoor PM_2.5_ within the disadvantaged city of Santa Ana before, during, and after the 4th of July; (2) identifying the indoor–outdoor PM_2.5_ ratio at co-located sampling sites to understand how firework-related emissions penetrate the indoor environment; and (3) analyzing the inter-city variability of July 4th PM_2.5_ levels between cities in Southern California, and how such levels correlate with inter-city differences in sociodemographic factors and firework legalization.

We hypothesized that Santa Ana would incur exceptionally high firework-related PM_2.5_ pollution, that the indoor environment would confer some level of protection from such pollution, and that cities characterized by greater proportions of low-income, Hispanic residents would experience greater July-4th-related air pollution on average compared with predominantly White, affluent communities. Furthermore, we hypothesized that cities in which street-level firework use is legal would experience greater firework-related air pollution on July 4th than communities where such activities are prohibited.

## 2. Methods

This study was conducted as part of a community–academic partnership involving the University of California, Irvine, and a local California-based non-profit called GREEN-MPNA. MPNA has been serving community members of southeast Santa Ana, California, for over 30 years. In 2012, MPNA founded GREEN-MPNA, the prefix of which stands for Getting Residents Engaged in Empowering Neighborhoods. GREEN-MPNA and its programs emerged from the specific needs identified by Madison Park residents, including support for youth and families regarding access to educational and leadership opportunities as well the needs to improve health outcomes through health education and the establishment of a safe and clean environment. Led by residents of southeast Santa Ana, GREEN-MPNA in recent years has been studying environmental justice and related health risks associated with air pollution facing local residents. Given the legal use of street-level fireworks in Santa Ana, along with the widespread use of illegal fireworks, the 4th of July holiday celebration in Santa Ana is known by locals to create relentless noise disturbances, light pollution, and thick and persistent smoke which has led many residents to relate the experience to a “war zone.” Measuring firework-related air pollution is therefore a key interest among Santa Ana residents and GREEN-MPNA.

### 2.1. Study Region

Santa Ana is a densely populated city located in southern California in the southwestern region of the United States. It is the administrative center of Orange County, which is the sixth most populated county in the U.S. With a total 2021 population of approximately 310,000 residents, Santa Ana spans an area of 70.6 km^2^ and includes 61 census tracts [49]. In terms of population, Santa Ana ranks the second largest city in Orange County, and is the eleventh largest city in the state of California [50]. The majority of Santa Ana residents identify as Latina/o/x (76.0%), followed by Asian (12.1%) and non-Hispanic White (8.5%), with a relatively high proportion (41.5%) of residents being immigrants [51]. The city includes 80,265 housing units and has a median household income of $72,406 (2020 dollars) [51]. A schematic showing the location of Santa Ana within the state of California is presented in Figure S1 of the supplementary materials.

### 2.2. Field Sampling

In the summer of 2022, a community air monitoring field effort was carried out on 22 separate days spanning the roughly one-month period from June 21st to July 22nd. Of the 22 days of measurement, 11 days and 10 days occurred pre- and post-July 4th, respectively. Each day of sampling included 24-h of 1-min averaged PM_2.5_ and temperature measurements conducted by trained community volunteers who were outfitted with AtmoTube Pro personal air pollution monitoring devices (AtmoTech, Inc., San Francisco, CA, USA) in order to measure outdoor PM_2.5_ concentrations and their corresponding measurement times. In total, 27 AtmoTubes were deployed in the field at the homes of Santa Ana residents, 17 of which were stationed in a fixed outdoor location and 10 of which were co-located in a fixed indoor location. This resulted in a total of 17 homes that contained an outdoor air monitor and 10 homes that contained both an outdoor and an indoor air monitor.

While reporting volatile organic compound (VOCs) measurements and multiple size fractions of particulate matter (PM), the AtmoTube Pro (henceforth, “AtmoTube”) is best suited to measure concentrations of PM_1_ and PM_2.5_ as well as temperature and humidity [52,53]. Equipped with an optical PM sensor, the AtmoTube measures PM using a measurement principal that is based on laser light scattering [54]. The AtmoTube has recently undergone field evaluation by the South Coast Air Quality Management District (SCAQMD) where it demonstrated a high measurement accuracy for the detection of ambient PM_1_ and PM_2.5_ concentrations when compared with federal equivalent method (FEM) instruments (R^2^ = 0.79–94) [52]. Given this, and since PM_2.5_ is a regulatory air pollutant that has been linked with numerous adverse health outcomes, measurements of PM_2.5_ are the focus of this study.

### 2.3. PurpleAir Network PM_2.5_ Data

The PurpleAir network is a worldwide network of low-cost PM_2.5_ monitors that began deployment in 2017. The latest model (PA- II-SD) contains two PMS5003 sensors (Plantower, Beijing, China), which estimate particle mass concentrations based on light scattering [55]. Overall, PurpleAir PA-II sensors show moderate to good accuracy, compared with reference PM_2.5_ measurements (i.e., R^2^ ~0.93 to 0.97), over a concentration range of 0 to 250 μg/m^3^ [56]. Further details regarding the lab evaluation of PurpleAir sensors conducted by the South Coast Air Quality Management District (AQ-SPEC team) and other research groups can be found elsewhere [57,58]. Prior studies have demonstrated the utility of using PurpleAir monitors to supplement regulatory monitors for PM_2.5_ exposure assessment [59]. In the present study, 10-min averaged PM_2.5_ concentration data and temperature data during the months of June and July, 2022, were retrieved from the PurpleAir network using the ThingSpeak’s API provided by the PurpleAir company [60]. Specifically, we extracted both indoor and outdoor measurements across the six most southern counties of California including Los Angeles, Orange, San Bernardino, Imperial, San Diego and Riverside counties. Of note, Imperial County, which included data from only a single sensor in one of its seven cities, was excluded from this analysis.

In order to reduce the impacts of potential sensor malfunction, intra-sensor bias, and other environmental and operational parameter impacts, all PurpleAir measurements underwent a two-step pre-processing procedure that included both quality control and calibration. The quality control procedure included the following four steps, which are similar to those described elsewhere [61,62].

1. Removal of malfunctioning sensor data based on a low frequency of change (5-day moving standard deviation of zero) in their reported measurements over time.
2. The setting of PM_2.5_ outliers that exceed the sensor’s effective measurement range (daily values > 500 μg/m^3^) to 500 μg/m^3^.
3. Identification of periods of prolonged interruption or data loss due to power outages or data communication loss using a 75% completeness criterion (≥108 10-min measurements in a day).
4. Examination of the correlation from dual-channel readings for each sensor within a given month of operation based on calculated statistical anomality detection indicators as the coefficient of determination R^2^ > 0.8 and mean absolute error < 5.

Approximately 2.8 million 10-min PM_2.5_ observations were available during the three-year study period, which was reduced to 2.4 million after applying the four steps described above.

### 2.4. Socioeconomic and Other Data

Socioeconomic data for each city within the five counties (Los Angeles, Orange, San Bernardino, San Diego and Riverside) were obtained via the 2020 American Community Survey and paired with city-level air pollution data. City data were used because firework-related policies are typically promulgated at the city level. Such firework legalization data for each city are obtained from the California Fireworks Newswire, which compiles information from the Office of the California State Fire Marshal, United States Fireworks Safety Commission, TNT Fireworks (American Promotional Events, Inc., Florence, AL 35631), and other relevant sources as it relates to the legal sale and use of household (or street-level) fireworks throughout the state. Cities where household level fireworks are legal for sale and use in southern California are presented in Table S1 of the supplemental materials. All calculations and statistical analyses performed in this study were carried out using SAS software [63].

### 2.5. Santa Ana Statistical Analysis

In order to carry out statistical and spatial analyses for the Santa Ana field monitoring, AtmoTube data and GPS data were merged using time stamps at the 1-min resolution. Since GPS measurements were recorded every 15 s, this required the conversion of GPS data into 1-min averages (i.e., averaging the latitude and longitude coordinates to yield an average location). Matched data were subsequently imported into ArcGIS version 10.8.1 software (ESRI, Redlands, CA, USA) for spatial analysis.

For statistical analysis, data were examined separately for the 24-h calendar days of July 4th and 5th, and also during the “24-h peak firework period” spanning July 4th (6AM) to July 5th (6AM) and during the “12-h peak firework period” spanning July 4th (6PM) to July 5th (6AM). Data were also analyzed according to the “pre-holiday period” (June 21st to July 3rd) and “post-holiday period” (July 5th to July 22nd) in order to evaluate potential differences in air pollution. For this analysis, weekend and weekday measurements were examined separately to avoid introducing a potential day-of-week bias. Given our interest in firework-related air pollution, which we anticipated to be highest on Friday and Saturday nights, our analysis defined weekdays (Mon–Thurs) and weekends (Fri and Sat) according to this expected variation. Since Sunday does not experience weekday traffic, nor was anticipated to experience typical weekend firework activity, Sunday was considered dissimilar to either group and was therefore excluded from analysis.

### 2.6. Inter-City Statistical Analysis

To expand our analysis for the comparison of cities across southern California, we used PurpleAir data combined with ArcGIS software. Of 191 incorporated cities from the five counties analyzed, 132 (69%) contained PM_2.5_ data on the 4th of July (after considering 75% completeness criteria). To compare peak outdoor PM_2.5_ levels across all cities, the PM_2.5_ average was calculated over the “24-h peak firework period” (as defined previously) for each city where at least one sensor existed. This also enabled a comparison with the peak firework period as calculated across AtmoTube measurements collected by Santa Ana residents.

Additionally, bivariate regression models examined the relationship between key socioeconomic factors and average PM_2.5_ during the “24-h peak firework period” at the city level. Multivariate regression analysis subsequently included all statistically significant terms (as determined from bivariate analysis) into individual models (one model for each county) and a pooled model (all counties combined). Backward stepwise elimination of non-significant terms was then applied to result in a more parsimonious model. Statistical significance was set at two-sided *p* = 0.05.

For outdoor analyses and visual mapping purposes, we also calculated a peak 24-h firework enrichment value to understand the elevation of July 4th-related air pollution relative to baseline concentrations. The result was a value called “PM_2.5_ enrichment.” Baseline was defined as the PM_2.5_ averaged over two similar 24-h periods (same 24-h window as the non-baseline 24-h peak), one week prior to the 4th of July.

For indoor/outdoor analysis of PM_2.5_ data, ArcGIS software was used to identify co-located indoor and outdoor PurpleAir sensors. A co-located sensor was defined as an outdoor sensor that was within a 500 m radius of an indoor sensor. Where multiple outdoor sensors existed near an indoor sensor, air pollution data was averaged across all co-located sensors. A sensitivity analysis defining co-located pairs to be within a 50 m radius demonstrated minimal change (±6%) in the final results.

## 3. Results

### 3.1. Santa Ana Analysis

**Figure 1.**
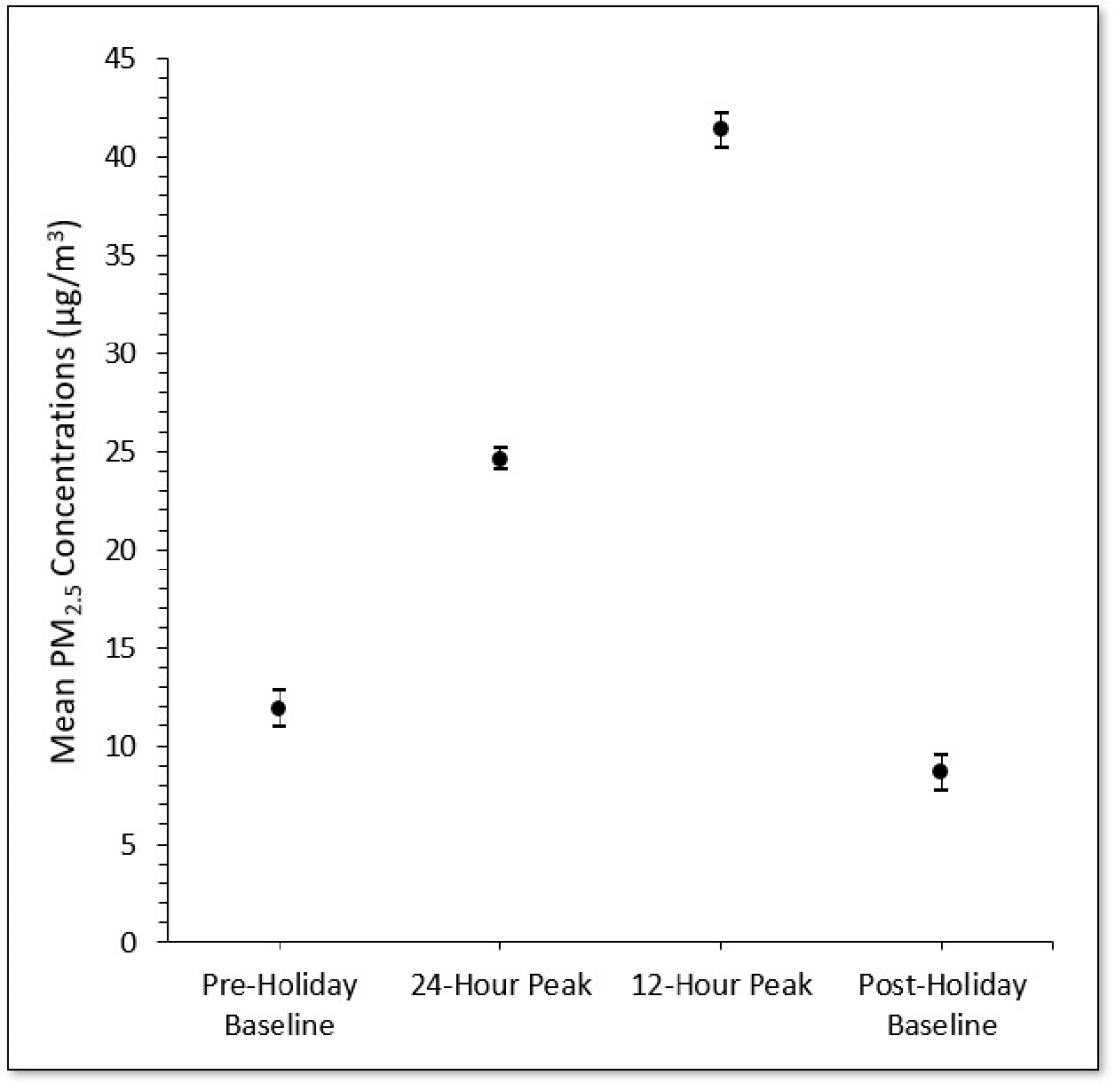
Average outdoor PM_2.5_ concentrations during the “24-h peak firework period” spanning July 4th (6AM) to July 5th (6AM) and during the “12-h peak firework period” spanning July 4th (6PM) to July 5th (6AM), relative to the non-holiday baseline periods.

Figure 1 presents results from 351,612 one-minute averaged PM_2.5_ measurements (5860 h) collected from June 21st to July 22nd using 17 outdoor AtmoTube sensors in the city of Santa Ana, CA, USA. As shown, the highest average outdoor PM_2.5_ concentration was that which occurred during the 12-h peak firework period (41.4 μg/m^3^), followed by the peak 24-h period (24.6 μg/m^3^), with the 95th percentile of outdoor PM_2.5_ being 113.1 μg/m^3^ during the 48-h July 4th and 5th period. By comparison, the average outdoor PM_2.5_ concentration measured during the non-holiday baseline period was 8.5 μg/m^3^, with a 95th percentile outdoor PM_2.5_ of 18.2 μg/m^3^. Relative to baseline, the average PM_2.5_ concentrations measured during the 12-h peak firework period and 24-h peak period were roughly five and three times greater, respectively.

When examining the diurnal pattern of average PM_2.5_ concentrations across all air monitors for each of the 22 days of field monitoring, the majority of hourly measurements exhibited a variability that ranged from approximately 5 to 20 μg/m^3^. Overwhelmingly, the highest peak occurred post-sunset on July 4th, exhibiting hourly average PM_2.5_ concentrations above 160 μg/m^3^ for two consecutive hours from 9PM to 11PM, and which remained above baseline until approximately 2AM. With the exception of episodic peaks, the diurnal pattern of PM_2.5_ pollution tended to exhibit a minimum in the mid-to-late afternoon accompanied by an overnight maximum, with overnight levels that tended to be substantially higher during the pre-holiday period compared with the post-holiday period. A time-series plot of hourly average PM_2.5_ concentrations for each day of air monitoring is presented in Figure S2 of the supplemental materials.

When comparing average PM_2.5_ concentrations measured across 10 sites in Santa Ana using co-located indoor and outdoor sensors during the July 4th holiday period, outdoor PM_2.5_ levels were found to be substantially higher for six of the ten sites. The indoor-to-outdoor (I/O) PM_2.5_ concentration ratio ranged from 0.09 to 1.4 across the 10 sites, with an overall mean and median of 0.8 and 0.76, respectively. Table 1 presents similar results, albeit eliminating one sensor (n = 9) that was an outlier for which no baseline data existed for comparison. A graph depicting the relative average indoor and outdoor PM_2.5_ concentrations by sensor ID are presented in Figure S3 of the Supplementary Materials.

**Figure 2.**
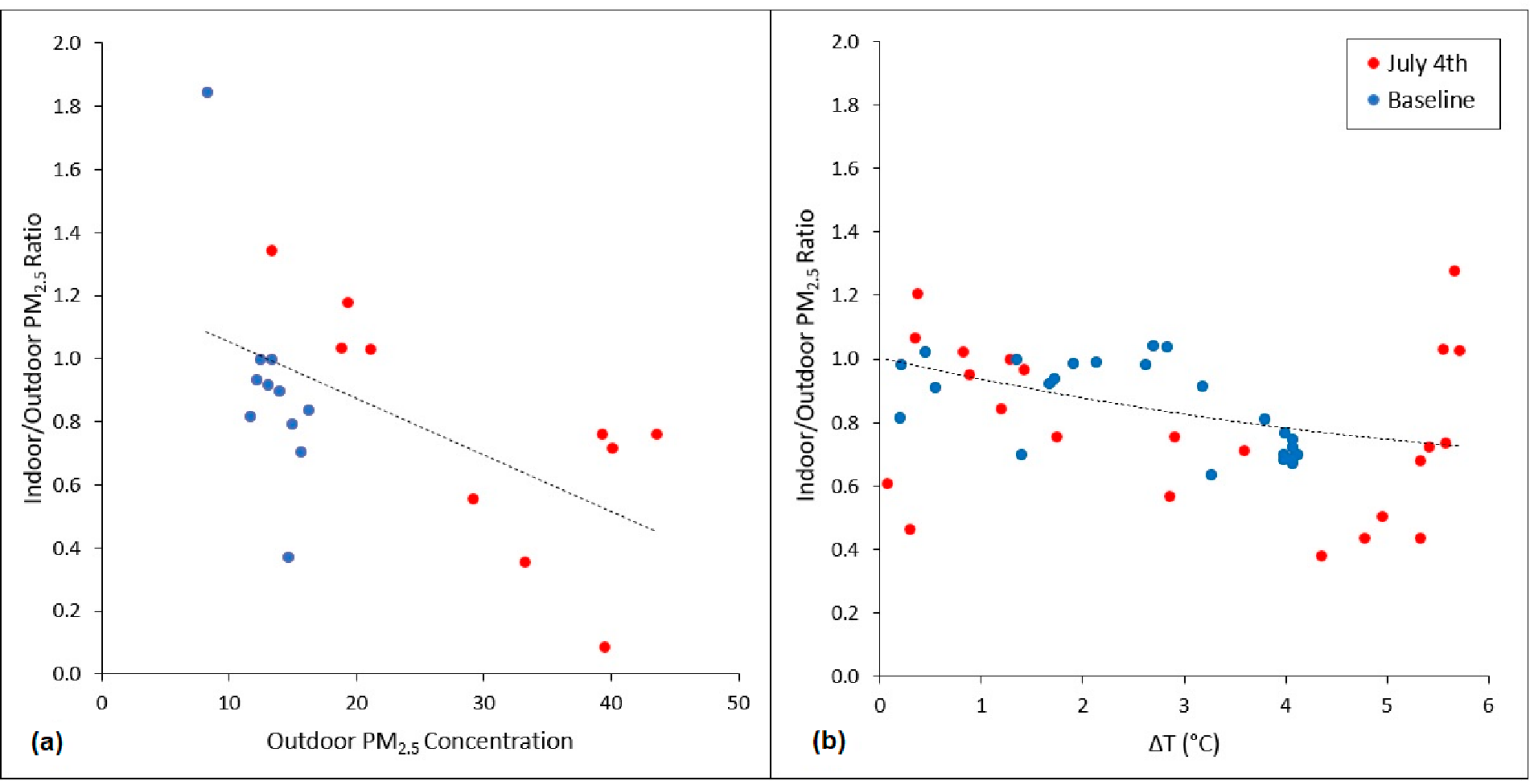
(**a**) Average 24-h I/O PM_2.5_ concentration ratios plotted against outdoor PM_2.5_ concentrations and (**b**) hourly average I/O PM_2.5_ ratios plotted against the difference between outdoor and indoor air temperatures (ΔT) for 10 co-located AtmoTube devices during the 24-h peak firework period on July 4th and during the baseline period.

Figure 2a presents a scatter plot of the I/O ratios for 10 co-located AtmoTube devices during the 24-h peak firework period on July 4th and during the baseline period. An overall negative correlation was observed with increasing outdoor PM_2.5_ concentrations. Figure 2b shows the hourly I/O ratios average across all co-located sensors on the y-axis and the difference between the outdoor and indoor temperature on the x-axis, for the same two time periods. A negative, curvilinear correlation was observed, showing that higher differences between outdoor and indoor temperatures tended to yield lower I/O ratios.

### 3.2. Inter-City Analysis

**Figure 3.**
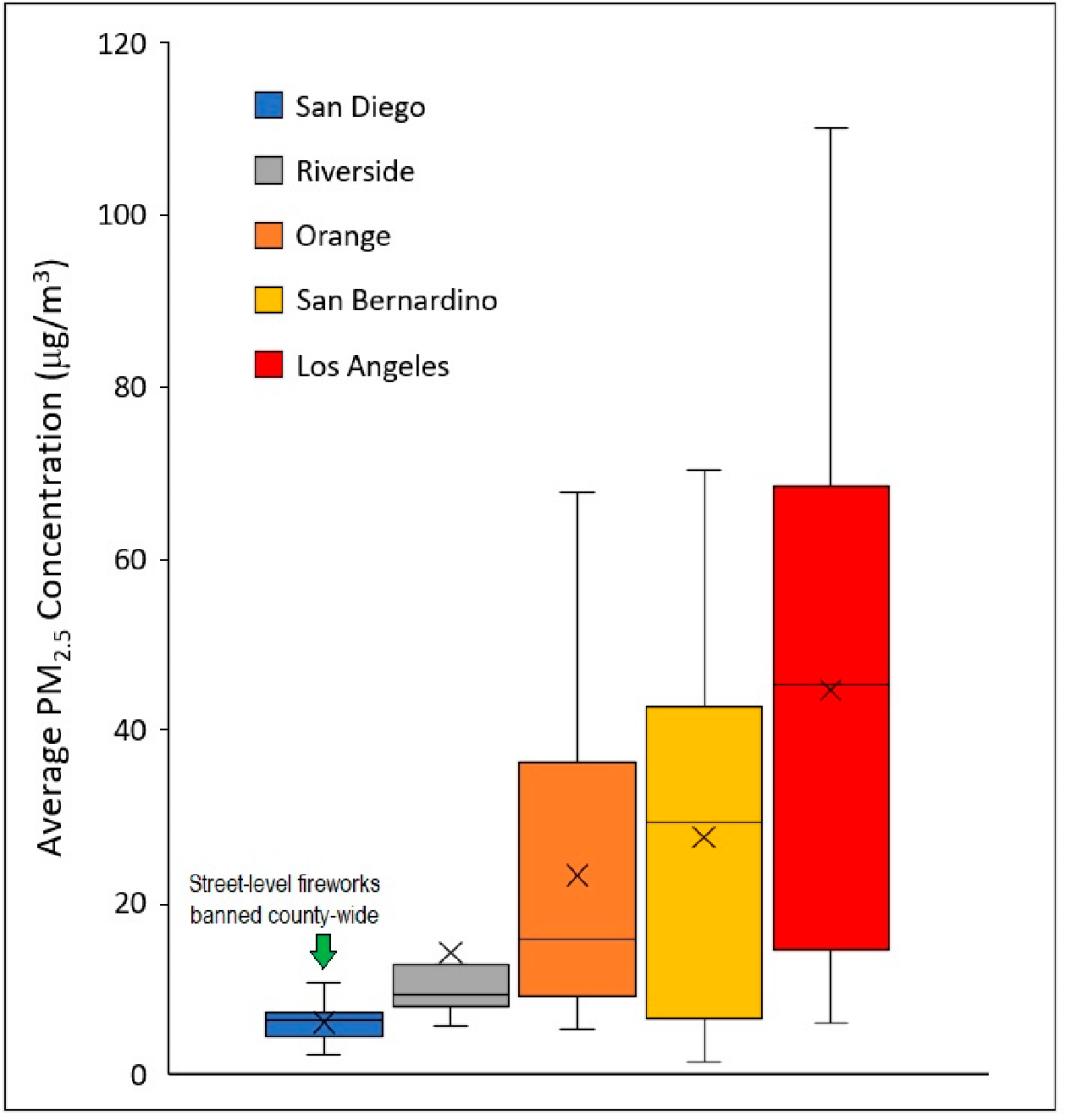
Boxplots depicting county-level PM_2.5_ concentrations during the 24-h peak firework period on July 4th and 5th. The lower and upper boundaries of each box indicate the interquartile range (IQR) of the sample, while the centerline and “X” symbol indicate the sample median and mean, respectively. The lower and upper whiskers indicate the minimum and maximum data points after excluding outliers as defined as Q_1_ or Q_3_ ± 1.5*IQR.

Figure 3 presents boxplots depicting county-level PM_2.5_ concentrations (calculated using city averages) during the 24-h peak firework period on July 4th and 5th. Overall, average PM_2.5_ concentrations at the county level were greatest for Los Angeles (44.6 μg/m^3^), followed by San Bernardino (27.5 μg/m^3^). Mean concentrations were approximately the same (±5%) as the medians for these cities, suggesting that the data were not skewed by outliers. These cities also exhibited the greatest inter-city average PM_2.5_ variability, where average PM_2.5_ concentrations in the 90th percentile of the most polluted cities were roughly 15 times greater than the least polluted city. Relative to the non-holiday baseline, the percent increase in average PM_2.5_ concentrations during the peak 24-h firework period was also greatest for Los Angeles (3.3-times above baseline) and San Bernardino (2.4-times above baseline) counties. The individual city with the highest average 24-h PM_2.5_ concentration was San Gabriel (110.1 μg/m^3^), located in Los Angeles County. The top 10 cities with the highest average 24-h PM_2.5_ concentration were also located in Los Angeles. The county with the overwhelmingly lowest average PM_2.5_ concentration (5.9 μg/m^3^) was San Diego, where street-level fireworks are prohibited.

Table 2 presents results following multiple regression analyses in which significantly correlated terms (as reported in Table S2) were used to develop multivariate models for each county and for all counties combined in order to understand the combined effects of multiple predicters. Results demonstrated race/ethnicity to be the only significant predictors of July 4th air pollution in the counties of Los Angeles, Orange, and Riverside, with higher air pollution being associated with greater proportions of Hispanic residents and/or lower proportions of White residents. These terms alone explain 21% to 51% of the variance in air pollution (Los Angeles, R^2^ = 0.21; Orange, R^2^ = 0.51; Riverside, R^2^ = 0.37). In San Bernardino, population density was the overwhelming predictor of PM_2.5_, with higher density being associated with more pollution (R^2^ = 0.80). Regression plots of these terms for each county are presented in Figure S4 of the Supplemental Materials. When combining city-level data from all counties, a greater proportion of foreign-born residents was associated with higher PM_2.5_ concentrations. When substituting this term for the “percent White” or “percent Hispanic” variables, these terms were also statistically significant, showing the same directions as those reported previously.

**Figure 4.**
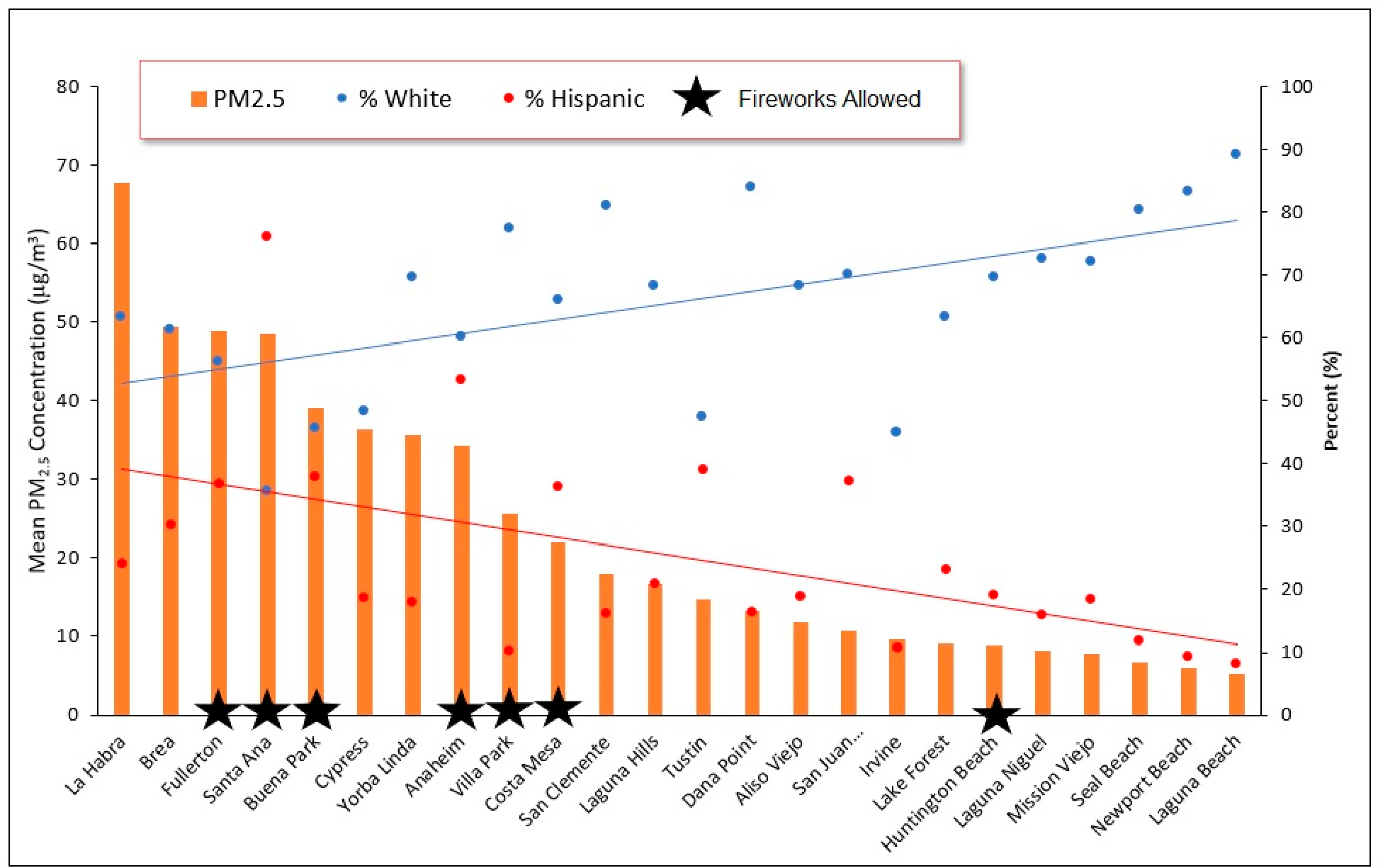
Inter-city comparison of average PM_2.5_ concentrations using data collected by the PurpleAir monitoring network in Orange County during the 24-h peak firework period spanning July 4th (6AM) to July 5th (6AM) overlaid with race/ethnicity data.

Figure 4 presents an inter-city comparison of average PM_2.5_ concentrations using data collected by the PurpleAir monitoring network during the 24-h peak firework period (as defined previously) overlaid with race/ethnicity data for Orange County, which is the county for which such disparities were of greatest interest to community partners and for which the highest correlation coefficients existed between PM_2.5_ and socioeconomic factors. As demonstrated, among the 24 of 34 Orange County cities for which July 4th data were available, Santa Ana ranked fourth in terms of the highest 24-h average PM_2.5_ concentrations during July 4th, with La Habra, Brea and Fullerton ranking first, second, and third, respectively. Variability in the 24-h average PM_2.5_ concentration was extremely high, with the top five most polluted cities exhibiting an average PM_2.5_ level (50.7 μg/m^3^) nearly eight-times higher than the average of the five least polluted cities (6.8 μg/m^3^), and the top city (La Habra) showing a 24-h average PM_2.5_ level (67.7 μg/m^3^) that was 13-times greater than that of the least polluted city (Laguna Beach, 5.3 μg/m^3^).

Of the 10 cities with the highest average PM_2.5_ concentrations in Orange County, the majority (six of ten) were those within which state-approved street fireworks were permitted for sale and use, compared with just a single such city when examining the 10 least polluted cities. On average, the five cities with the highest July 4th PM_2.5_ pollution also tended to be composed of a lower proportion of White residents (52.3%) and higher proportions of Hispanic residents (41.0%) compared with the five cities with the lowest PM_2.5_ levels, which were predominantly White (79%) with a low percent Hispanic population (16.5%). On average, the five least polluted cities were also characterized by a 14% higher median household income, compared with the five most polluted cities. Three of the four least-polluted cities included “beach cities” (Seal Beach, Newport Beach, and Laguna Beach) characterized by higher affluence and an ocean breeze influence.

**Figure 5.**
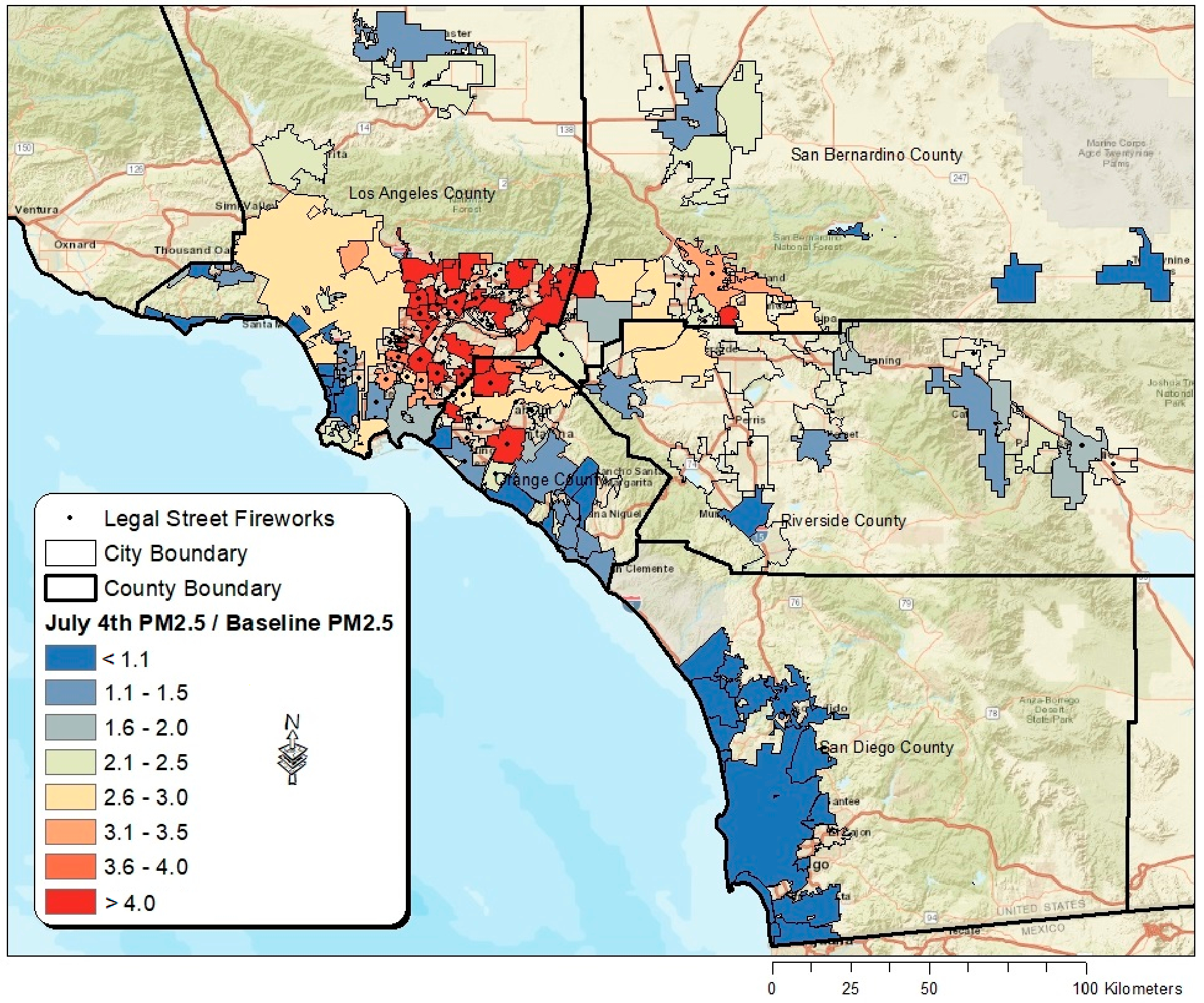
Map showing average PM_25_ concentrations on July 4th divided by baseline PM_25_ across incorporated southern California cities where PurpleAir data were available, along with the cities where street-level fireworks are permitted for sale and use.

Figure 5 depicts a map showing average PM_25_ concentrations during the peak 24-h firework period divided by baseline PM_25_ across incorporated southern California cities where PurpleAir data were available. Additionally, the cities in which street-level fireworks were permitted for sale and as of 2022 are shown. High variability exists when comparing cities and counties in terms of July 4th PM_2.5_ concentrations relative to baseline. In Los Angeles, many inland cities exhibited July 4th averages that were over four-times that of the baseline measurements. In general, coastal cities tended to experience less air pollution than inland cities, while San Diego County (where street-level fireworks are not permitted in any city) experienced the least air pollution. On average, cities where street-level fireworks are legal experienced roughly 47.3% higher PM_2.5_ concentrations (40.5 μg/m^3^) on July 4th compared with cities where such fireworks are prohibited (27.5 μg/m^3^).

**Figure 6.**
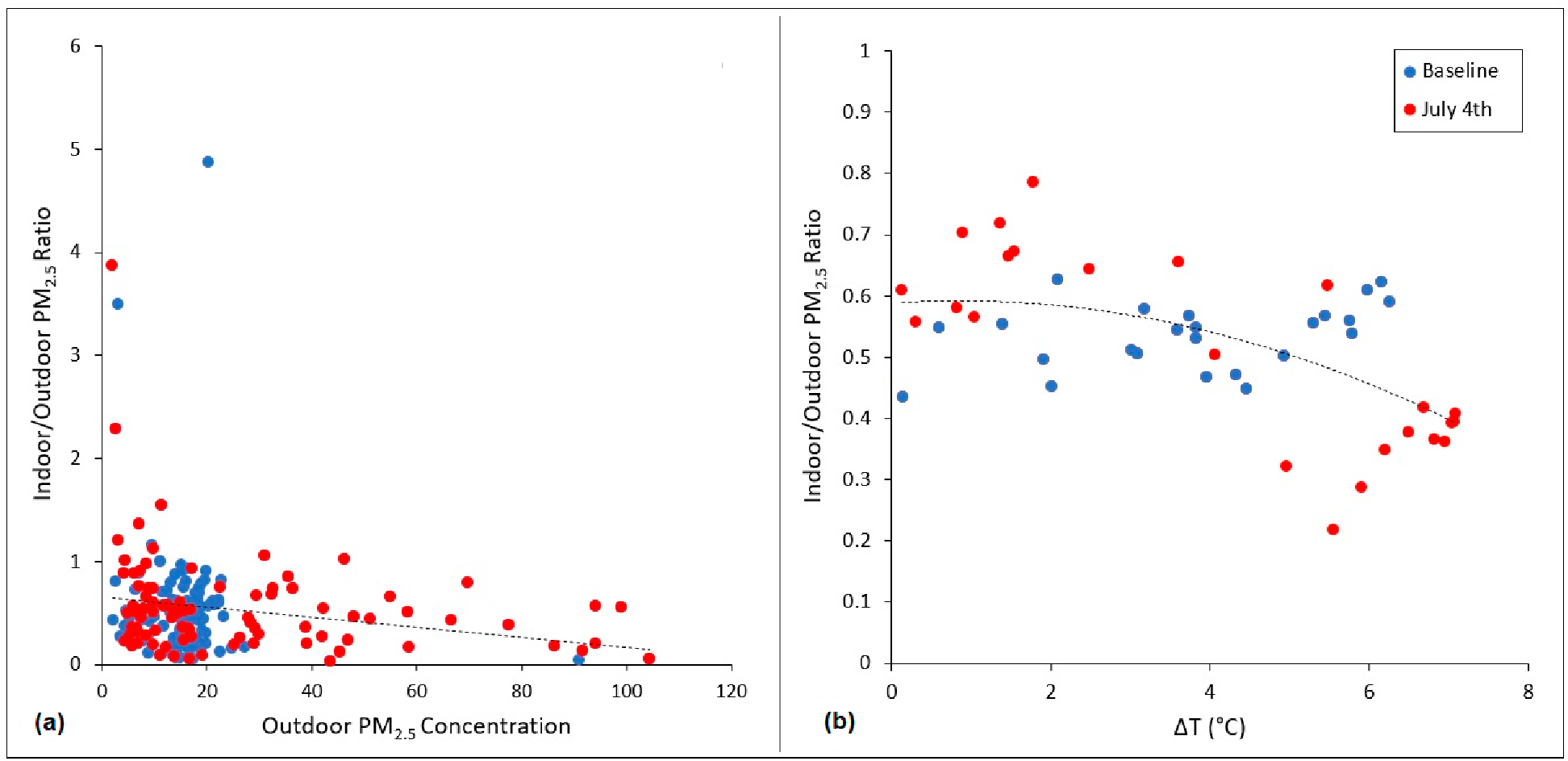
(**a**) Average 24-h I/O ratios plotted against outdoor PM_2.5_ concentrations and (**b**) hourly average I/O PM_2.5_ ratios plotted against the difference between outdoor and indoor air temperatures (ΔT) across 92 co-located PurpleAir sensors during the 24-h peak firework period on July 4th and during the baseline period.

Figure 6a presents a scatter plot of the I/O ratios for 92 co-located PurpleAir devices during the 24-h peak firework period on July 4th and during the baseline period. An overall negative correlation was observed with increasing outdoor PM_2.5_ concentrations. Figure 6b shows the hourly I/O ratios average across all co-located sensors on the y-axis and the difference between the outdoor and indoor temperature on the x-axis, for the same two time periods. A negative, curvilinear correlation is observed, showing that higher differences between outdoor and indoor temperatures tended to yield lower I/O ratios. This was most distinct for measurements collected on July 4th, whereas baseline samples yielded little trend.

Finally, Table 3 presents summary statistics of the I/O PM_2.5_ ratio during the peak 24-h firework period as measured by PurpleAir devices across southern California. As shown, the average (0.56–0.63) and median (0.47–0.49) ratios calculated across PurpleAir sensors throughout southern California generally agreed with one another when comparing intercity statistics with those of co-located monitors. Both the mean and median I/O baseline PM_2.5_ ratios were very similar to their non-baseline data ratios (±0.1).

## 4. Discussion

This study presents findings from a community-based air monitoring campaign that engaged citizen scientists for the measurement of indoor and outdoor PM_2.5_ concentrations using low-cost sensors before, during, and after the 4th of July firework holiday celebration in Santa Ana, California, as well as findings from an analysis of regional PM_2.5_ data collected by the PurpleAir network across southern California. In general, the average PM_2.5_ concentration measured outdoors during the 4th of July was roughly three-to-five times higher than baseline, with average hourly concentrations exceeding 160 μg/m^3^. These results are similar to prior research demonstrating firework-related air pollution to contribute heavily to total PM_2.5_ on the 4th of July holiday [27,36,64]. Importantly, however, our findings also demonstrate that outdoor July 4th air pollution is be roughly two-times higher than that measured in the indoor environment using co-located monitors, and to be mostly predicted by household firework legalization and racial/ethnic characteristics at the city level.

### 4.1. Santa Ana Analysis

Following an examination of hourly PM_2.5_ concentrations averaged across all air monitors for each day of the Santa Ana monitoring, variability was found to range considerably, with episodic hourly peaks that may reflect industrial activity, daytime firework use, or secondary particle formation, whereas the peaks occurring after sunset were likely due to nighttime firework use. During community meetings leading up to the field campaign, Santa Ana residents indeed confirmed extensive nighttime firework activity that regularly occurred in the weeks prior to July 4th. Overwhelmingly, the highest peak PM_2.5_ concentrations occurred after sunset on July 4th, corresponding with the occurrence of municipal firework shows and the widespread use of household-level fireworks by residents.

The diurnal pattern of PM_2.5_ pollution tended to exhibit a minimum in the mid-to-late afternoon accompanied by an overnight maximum. This was the case for both weekdays and weekends during both the pre- and post-July 4th periods. That overnight levels were substantially higher during the pre-holiday period compared with post-holiday, and during the weekends compared with weekdays, suggests that the overnight increase in air pollution may be related to the use of street-level fireworks (including illegal varieties) in the days and weeks leading up to July 4th in Santa Ana, thus affirming community observations.

An analysis of indoor and outdoor air pollution across co-located sites in Santa Ana during July 4th showed outdoor levels to be about 30% higher, thus serving as evidence that staying indoors affords residents some level of protection against firework-related outdoor air pollution exposure. Nonetheless, wide variability in the I/O ratio existed. Factors that may explain such variability include the structural characteristics of the building where PM_2.5_ sensors were placed (e.g., the age of the building may influence building porosity and outdoor air penetration), the way in which the building was used during the July 4th monitoring period (e.g., windows open or closed, frequency of doors being opened, etc.), as well as the behavior of room occupants (i.e., whether smoking and/or cooking occurred indoors).

On average, the I/O PM_2.5_ ratio was shown to decrease at greater outdoor air pollution levels. This may serve as evidence that residents closed their windows and doors as outdoor air pollution and/or noise levels increased (as opposed to taking such actions due to the onset of dark, bedtime, and/or cooler nighttime temperatures). This hypothesis is affirmed by results showing the I/O ratio to decrease with increasing outdoor/indoor temperature differentials on July 4th, and less so on baseline days. In some cases, high I/O ratios coincided with high temperature differentials on July 4th, perhaps indicating the presence of indoor emissions sources (e.g., smoking or cooking).

### 4.2. Inter-City Analysis

To compare the level of firework pollution in the city of Santa Ana with that of the neighboring cities of Orange County and southern California, an additional analysis was carried out using publicly available PurpleAir sensor data. Countywide, we found Santa Ana to rank fourth out of 24 cities in terms of the highest average PM_2.5_ concentration measured during the peak 24-h firework period on July 4th. The average concentrations for Santa Ana were roughly two-times higher than that measured using AtmoTubes during community sampling, likely resulting from regional differences in the placement of sensors. In general, PM_2.5_ variability was very high between cities, with the top five most polluted cities in Orange County exhibiting nearly eight-times higher PM_2.5_ levels compared with the least contaminated cities. Across southern California, variability was twice as high. Los Angeles exhibited the highest July 4th PM_2.5_ average, which is consistent with findings from our prior work [36].

While reasons behind the variability in air pollution levels are multifarious, including factors such as local meteorology, we generally found that the cities with the highest PM_2.5_ pollution tended to be those within which street-level fireworks are legal for sale and use (or adjacent to such cities). In general, such cities had roughly 50% higher PM_2.5_ levels on July 4th. A county-wide analysis showed similar findings. For instance, in San Diego, where street-level fireworks are prohibited county-wide, average PM_2.5_ concentrations were three-to-eight times lower than the other four counties examined across southern California. This suggests that, while aerial firework shows contribute extensively to regional air pollution, the street-level use of fireworks may be more important to air pollution at the ground level where exposure is most relevant.

That street-level fireworks are major contributors to poor air quality on the 4th of July is consistent with our prior study, which showed July 4th-related PM_2.5_ concentrations in California to be over 50% higher in 2020 compared with 2019 despite the fact that municipal firework shows were largely canceled in 2020 due to the COVID-19 pandemic [36]. Furthermore, we found higher levels in southern California, where firework-related laws are generally more lax [65,66]. As California’s South Coast Air Quality Management District noted in 2020, the extensive use of personal fireworks produced a “*nonstop barrage of aerial bursts and explosions*” across southern California for hours [67]. This was accompanied by news reports that documented a statewide increase in the use of illegal household fireworks in 2020 leading up to the 4th of July [37–39].

Overlaying city-wide air pollution levels measured on July 4th in southern California with city-specific socioeconomic data demonstrated race/ethnicity to be the leading (and only) significant predictor of July 4th-related air pollution across three counties. Greater PM_2.5_ concentrations were associated with higher proportions of Hispanic residents and foreign-borne residents, and lower proportions of White residents. In one county, higher population density was the leading predictor. These findings are consistent with our prior research and underscore the importance of outreach and education about the deleterious effects of burning fireworks as well as related policies to curb emissions and improve environmental health equity [36].

While this study identifies socioeconomic disparities regarding firework-related policies and air pollution exposure on the 4th of July, identifying the reasons behind such disparities and the differences in city-by-city legalization of street-level fireworks is beyond the scope of the present paper. In terms of plausible hypotheses, however, one factor may be differences in cultural interest in fireworks and/or awareness of air pollution-related health effects, which may translate to differences in residential support for policies that either allow or ban street-level fireworks. Other factors may relate to the long history of systematic marginalization of low-income communities and communities of color that often leave such residents out of decision-making procedures relating to urban planning and local policy, resulting in such communities bearing disproportionate exposures to various environmental hazards.

Of note, in Orange County, cities with greater proportions of children and youth exhibited higher PM_2.5_ concentrations on average, which has important implications for public health among children, particularly given that short-term PM exposure can exacerbate asthma symptoms [68,69]. This correlation was not observed across other counties.

Analysis of co-located indoor and outdoor air pollution sensors using PurpleAir data demonstrated a similar pattern as that observed using AtmoTubes in Santa Ana. However, I/O ratios were appreciably lower, and the maximum temperature differential higher, when examining PurpleAir data. While it is possible that residents outside of Santa Ana merely closed their windows and doors more often on July 4th than those in Santa Ana (a small, unpublished survey indeed confirmed Santa Ana residents to generally leave their windows open at night), the discrepancy may also indicate newer and/or better insulated homes on average across the PurpleAir study domain compared with Santa Ana. Alternatively, or in conjunction, this may reflect less financial capacity to spend money on air conditioning in Santa Ana. These hypotheses are consistent with Santa Ana being both the oldest and lowest income city in Orange County.

Ways to ameliorate the problem of exceptionally high July 4th air pollution include increased outreach and awareness concerning the health effects of short-term air pollution exposure, along with the promulgation of relevant policy to protect vulnerable subgroups, which may include firework bans and/or increased enforcement of existing bans. Residents may also voluntarily opt to avoid purchasing and burning household fireworks in their communities, and to instead attend an existing firework display if desired. Solutions regarding municipal firework shows may include the relocation of certain shows from more vulnerable to less vulnerable areas and/or the reduction of the total number of fireworks used in each show and/or each city, the latter being akin to a cap-and-trade on total municipal firework burning. Of note, zero-emission alternative firework shows also exist, such as drone light shows, which have become increasingly popular in parts of China and the Middle East [70]. Additionally, research has shown that urban tree cover (especially low VOC emitters) can aid in the direct and indirect removal of air pollution (particularly when levels are high) through ambient temperature reduction, atmospheric deposition, and gaseous pollutant uptake [71].

Furthermore, this study has demonstrated that remaining indoors during firework episodes is an effective means of reducing PM_2.5_ pollution exposure, an action likely to be even more effective when paired with the use of an indoor air purifier. Through community workshops conducted in Santa Ana, this community–academic collaboration enabled residents to share concerns and insights about local air pollution, engage in discussions with experts, and observe firsthand the way in which firework activity increase measurable air pollution levels. As such, this study demonstrated community engagement as an effective means of raising awareness about air pollution exposure and mobilizing residents for the collection of local air quality information that fosters community empowerment and health education.

A primary strength of this study is the application of low-cost sensors using community-engaged research methods in order to produce a more spatially resolved understanding of regional air pollution exposure, including an inter-city comparison of PM_2.5_ variability, on the 4th of July. This is distinct from the recent literature, which has tended to employ readily available data from either 24 hour CSN stations [33] or scarcely distributed FEM monitoring stations [29] across California. Furthermore, in contrast to other studies that examine outdoor air pollution during firework celebrations, this study presents results from co-located indoor and outdoor monitors, therefore enabling an understanding of the extent to which the indoor environment may offer protection for residents against harmful firework-related exposures. Furthermore, this study examines how firework-related PM_2.5_ pollution at the city level is correlated with both socioeconomic factors and household firework legalization, therefore informing future policies related to health equity and environmental justice.

Limitations of this study include the absence of information relating to the building characteristics and at-home behavioral patterns surrounding indoor air monitoring. Additionally, this study did not consider meteorology nor its potential impact on the regional variation in PM_2.5_ concentrations. Furthermore, the PM_2.5_ averages calculated across each city, and within Santa Ana, were limited by the numbers of sensors available in each city. This may have introduced bias depending on where (and how many) monitors were placed within each city. What is more, since the PurpleAir network is still limited in regional distribution, not all cities within Orange County could be represented in our countywide analysis. Lastly, this study only characterized air pollution in the form of total PM_2.5_ and did not consider the chemical composition of particles nor gaseous pollutants. Therefore, it does not present a full picture of firework-related air pollution on the 4th of July.

## 5. Conclusions

This study presents results from a community-based air monitoring campaign that engaged citizen scientists for the measurement of indoor and outdoor PM_2.5_ concentrations using low-cost sensors before, during and after the 4th of July firework holiday celebration in Santa Ana, California, as well as findings from an analysis of regional PM_2.5_ data collected by the PurpleAir network across southern California. Results show average outdoor PM_2.5_ concentrations on July 4th to be three-to-five times higher than baseline, with hourly concentrations exceeding 160 μg/m^3^. Outdoor averages were roughly 30% to 100% higher than indoor levels. The most polluted cities exhibited 15-times higher PM_2.5_ levels compared with the least contaminated cities, and were often the cities where street-level fireworks were legal for sale and use. Race/ethnicity was found to be the leading predictor of July 4th-related air pollution across three counties in southern California, with greater PM_2.5_ being associated with higher proportions of Hispanic residents and lower proportions of White residents. Findings from this study underscore the importance of environmental justice as it relates to firework-related air pollution exposure, and the critical role city- and county-level firework policies play in determining such exposure.

## Supplementary Material

Supplementary Material

## Figures and Tables

**Table 1.** PM_2.5_ I/O ratio during the “24-h peak firework period” as measured using AtmoTube devices in Santa Ana, CA.

	Indoor PM_2.5_ / Outdoor PM_2.5_				
	Mean	S.D	Median	Min.	Max.

AtmoTube (Santa Ana)					
Baseline	0.91	0.31	0.91	0.71	1.9
Located Average (n = 10)	0.77	0.34	0.77	0.36	1.4

**Table 2.** Effect estimates (EE) and p-values following multivariate regression analysis using key outcome variables.

	ALL (n = 113)		Los Angeles (n = 60)		Orange (n = 24)		Riverside (n = 11)		San Bernardino (n = 18)	

	E.E.	*p*-Value	E.E.	*p*-Value	E.E.	*p*-Value	E.E.	*p*-Value	E.E.	*p*-Value

Intercept	0.33	0.95	**63.3**	**<0.01**	**55.3**	**<0.01**	−6.7	0.50	−1.85	0.67
%<Age18					0.39	0.74				
% White (non-Hispanic)			**−0.54**	**<0.01**	−**0.63**	**<0.01**				
% Hispanic							**0.45**	**0.049**		
% Foreign Born	**1.30**	**<0.01**							0.46	0.55
Per Capita Income	0.0	0.18	0.0	0.19	0.0	0.30	0.0	0.63		
Median Home Value									0.0	0.90
% College Educated			0.73	0.10	−0.61	0.11				
% High School Educated	0.32	0.39								
Population Density	0.0	0.67			0.0	0.54	0.0	0.82	**0.01**	**<0.01**

Notes: Bold indicates statistical significance (*p* < 0.05). Values for the intercepts and significant terms reflect final statistics following stepwise elimination of non-significant terms. San Diego County not included due to household-level fireworks not being permitted for sale and use. Imperial County not included due to existence of only one city measurement.

**Table 3.** PM_2.5_ I/O ratio during the “24-h peak firework period” as measured using PurpleAir devices across southern California.

	Indoor PM_2.5_ / Outdoor PM_2.5_				

	Mean	S.D.	Median	Min.	Max.

**PurpleAir (Southern California)**					
Baseline	0.63	0.72	0.47	0.14	5.2
Inter-City average (n = 57)	0.61	0.45	0.48	0.10	2.3
Baseline	0.56	0.61	0.47	0.05	4.9
Co-Located average (n = 90)	0.56	0.50	0.49	0.04	3.9

## Data Availability

All PurpleAir data used in this study is publicly available and can be accessed online (https://www2.purpleair.com/, accessed on 15 December 2020) while all community-collected air pollution measurements can be made available upon request from the authors.
