# Supplementary material for: Race and Street-Level Firework Legalization as Primary Determinants of July 4th Air Pollution across Southern California
Source: Atmosphere (Basel). Author manuscript; Available in PMC 2024 Sep 12. (PMC11392046; doi:10.3390/atmos14020401)
Supplement: Supplementary Material [file NIHMS1967182-supplement-Supplementary_Material.pdf]

Supplemental Materials

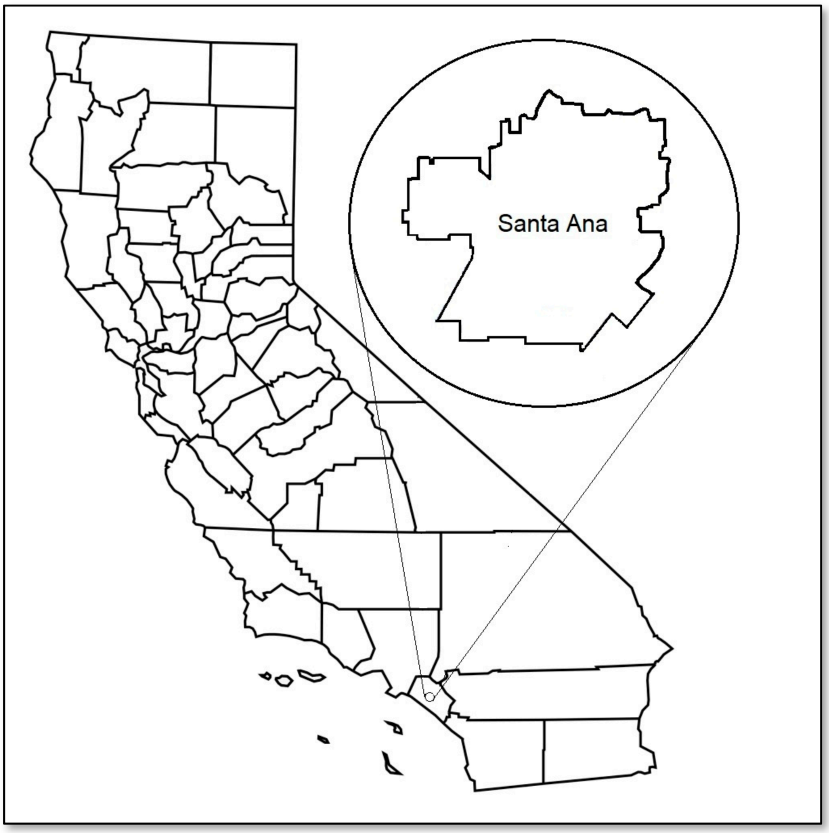

Figure S1. Map depicting city of Santa Ana within California.

Table S1. Southern California cities in which household fireworks are legal for sale and use.

County	City

Los Angeles	Alhambra, Baldwin Park, Bellflower, Carson, Compton, Downey, El Monte, Hawthorne, Inglewood, La Mirada, Lakewood, Lawndale, Maywood, Montebello, Monterey Park, Norwalk, Palmdale, Paramount, Rosemead, South Gate
Orange County	Anaheim, Buena Park, Costa Mesa, Fullerton, Huntington Beach, Santa Ana, Villa Park
Riverside	Indio
San Bernardino	Barstow, Chino Hills, Colton, Fontana, San Bernardino
San Diego	N/A

Figure S2 presents the diurnal pattern of average hourly $PM_{2.5}$ concentrations across all air monitors for each of the 22 days of field monitoring, with data collected during the pre- and post-holiday periods denoted in red and blue, respectively. Of note, the days shown in the figure have been offset by 6 hours to correspond with the 24-hour peak firework period, as defined previously, so as to ease visual interpretation of potential nighttime firework pollution. Data collected on the 4th of July is denoted in black. As shown, the majority of hourly measurements exhibited variability that ranged from approximately 5 to 20 μg/m^3.

Numerous hourly peaks ranging from approximately 20 to 40 μg/m^3 can be observed during the day from 10AM to 1PM, potentially reflecting non-firework related industrial or backyard barbecuing activity, or potential daytime firework use, whereas numerous other peaks occurred after sunset (8:08 PM). Overwhelmingly, the highest peak occurred post-sunset on July 4th, exhibiting hourly average $PM_{2.5}$ concentrations above 160 μg/m^3 for two consecutive hours from 9 to 11PM, which remained above baseline until approximately 2AM. With the exception of episodic peaks, the diurnal pattern of $PM_{2.5}$ pollution tended to exhibit a minimum in the mid-to-late afternoon accompanied by an overnight maximum. As can be seen, overnight levels tended to be substantially higher during the pre-holiday period compared to post-holiday.

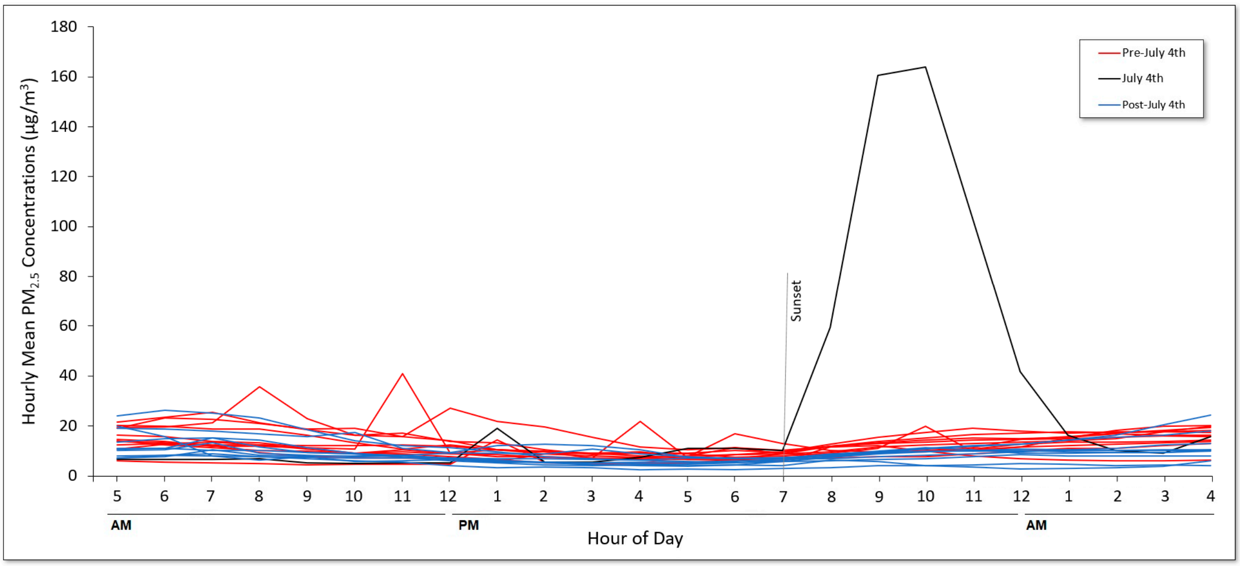

Figure S2. Hourly $PM_{2.5}$ concentration for each of 22 separate measurement days. Of note, days are defined as 5PM to 5AM so as to ease visual interpretation of potential overnight firework pollution.

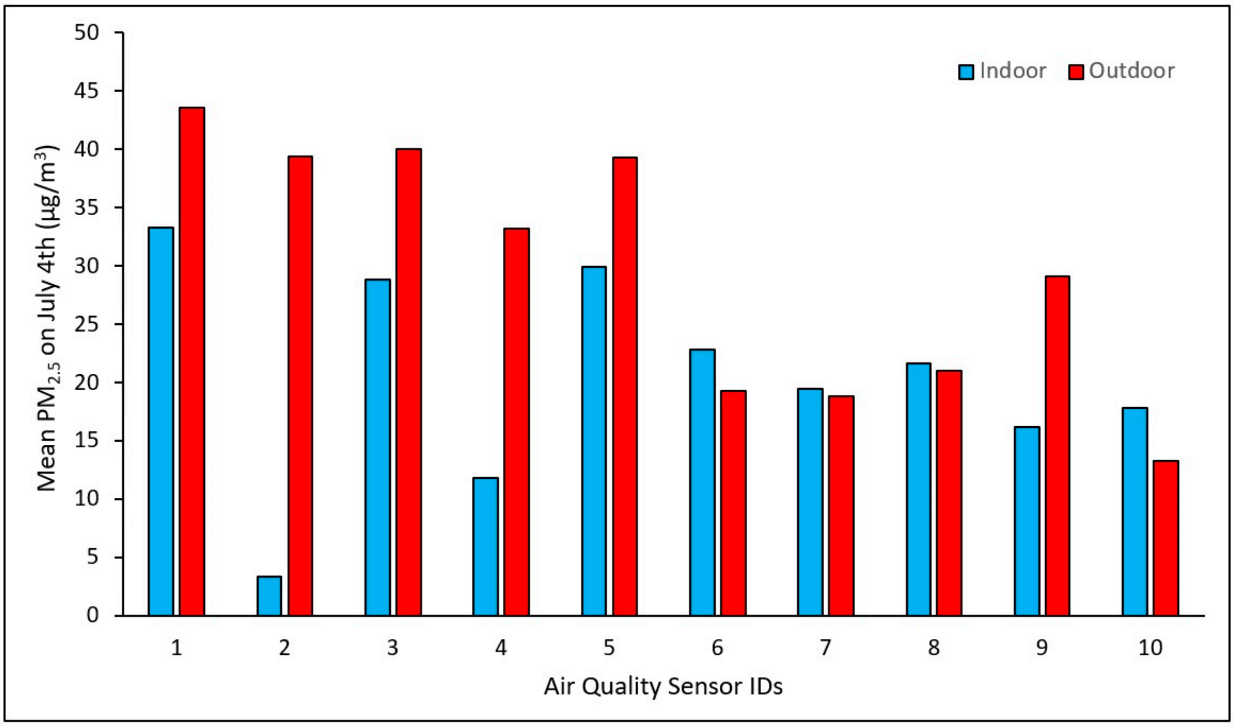

Figure S3. Comparison of average $PM_{2.5}$ concentrations measured across 10 sites in Santa Ana using co-located indoor and outdoor sensors during the peak 24-hour firework period.

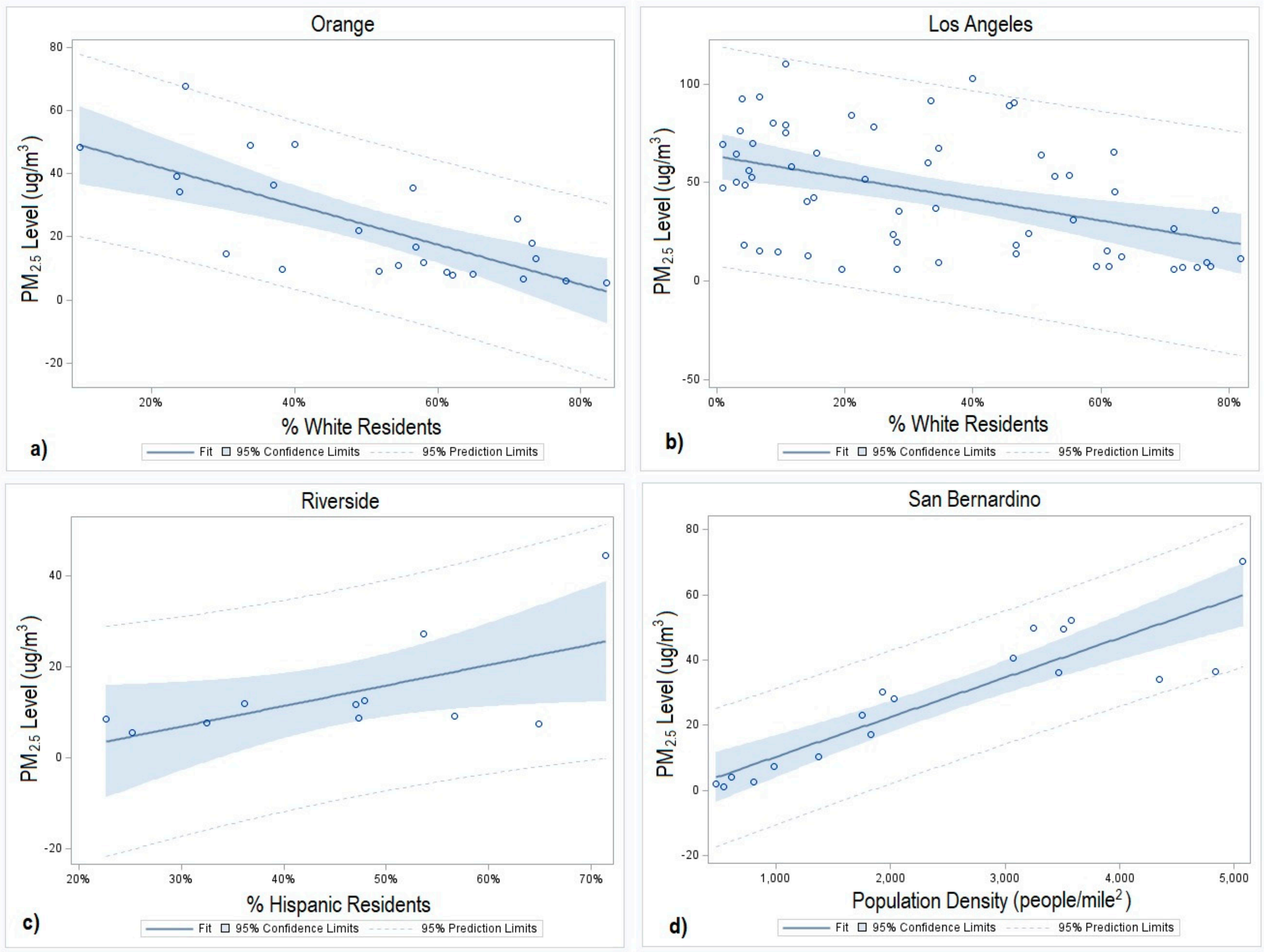

Figure S4. Regression plots of significant terms following multiple regression analysis by county (only one term remained significant per county following backward stepwise elimination procedure).

Table S2 presents Pearson correlation coefficients when examining scatter plots of city-average $PM_{2.5}$ concentrations measured during the peak 24-hour firework period and city-wide socioeconomic characteristics for four southern California counties where household-level fireworks were permitted for sale and use in 2022 (excluding Imperial County due to existence of only one city measurement), with bold indicating correlation coefficients ≥ 0.3 and statistical significance ($p<0.05$). Also shown (right column under each

county), are the correlation coefficients when plotting the same socioeconomic statistics against July 4th $PM_{2.5}$ Enrichment (24-hour Peak $PM_{2.5}$ / 24-hour Baseline $PM_{2.5}$).

In general, within counties, cities with higher proportions of White residents tended to have lower average $PM_{2.5}$ in terms of both absolute concentrations and relative enrichment during the peak 24-hour firework period, while the opposite $PM_{2.5}$ trend was apparent when examining the proportion of Hispanic and foreign-born residents ($p<0.05$). Economic indicators such as median household income, per capita income and median home value were generally negatively correlated, and poverty rate positively correlated, with July 4th $PM_{2.5}$ pollution at the city level across Los Angeles County and Orange County, whereas such correlations were either low or not statistically significant in Riverside County. In Orange and Riverside counties, the proportion of elderly residents (age > 65) within cities was moderately inversely correlated with absolute $PM_{2.5}$ concentrations during the holiday period, albeit not statistically significant only in Riverside. Additionally, Orange County showed higher proportions of youth and children in cities with higher $PM_{2.5}$ pollution. Patterns of correlation and statistical significance similar to those just describe were also observed for the race/ethnic, foreign-born, and per capita income terms when combining all cities into a single dataset (n=114).

Table S2. Pearson correlation coefficients when comparing the socioeconomic characteristics of each city with both its average $PM_{2.5}$ concentration measured during the peak 24-hour firework period (Peak $PM_{2.5}$) and its Peak $PM_{2.5}$-to-baseline ratio for four southern California counties.

	**July 4th Peak $PM_{2.5}$ | July 4th $PM_{2.5}$ Enrichment[a]**									
	**ALL (n=113)**		**Los Angeles (n=60)**		**Orange (n=24)**		Riverside (n=11)		San Bernardino (n=18)	
**% < Age 5**	**0.02**	**0.04**	**0.08**	**0.11**	**0.35**	0.37	0.41	-0.03	-0.29	-0.25
% < Age 18	-0.06	-0.02	-0.07	0.00	**0.49**	**0.54**	0.42	-0.04	-0.28	-0.31
% > Age 65	-0.16	-0.14	0.02	0.00	-0.37	**-0.41**	-0.52	-0.13	-0.11	0.05
% White (non-Hispanic)	**-0.49**	**-0.52**	**-0.45**	**-0.50**	**-0.72**	**-0.75**	-0.59	-0.30	**-0.49**	-0.33
% Hispanic	**0.30**	**0.34**	**0.31**	**0.38**	**0.69**	**0.65**	**0.61**	0.42	0.38	0.19
% Foreign Born	**0.53**	**0.51**	**0.48**	**0.47**	**0.46**	**0.53**	0.44	0.43	**0.64**	**0.52**
Median Household Income	-0.16	-0.21	**-0.37**	**-0.39**	-0.39	-0.39	0.32	0.10	0.38	0.39
Per Capita Income	-0.29	**-0.35**	**-0.43**	**-0.48**	**-0.60**	**-0.63**	-0.40	-0.10	0.21	0.28

% Poverty	-0.01	0.03	0.18	0.20	**0.45**	**0.46**	-0.23	0.02	-0.45	**-0.47**
Median Home Value	-0.10	-0.19	**-0.33**	**-0.41**	-0.39	**-0.41**	0.21	0.10	**0.54**	**0.53**
% College Educated	-0.19	-0.26	**-0.33**	**-0.41**	**-0.65**	**-0.63**	-0.42	-0.14	0.28	0.37
% High School Educated	**-0.31**	**-0.34**	**-0.31**	**-0.35**	**-0.53**	**-0.55**	-0.64	-0.46	-0.21	-0.04
Population Density	**0.30**	0.27	0.07	0.06	**0.51**	**0.55**	0.24	-0.04	**0.90**	**0.77**

[a] July 4th $PM_{2.5}$ Enrichment = 24-hour Peak $PM_{2.5}$ / 24-hour Baseline

Notes: Bold indicates correlation coefficients ≥ 0.3 and statistical significance ($p<0.05$). San Diego County not included due to household-level fireworks not being permitted for sale and use. Imperial County not included due to existence of only one city measurement.
